# Clustering Using Boosted Constrained k-Means Algorithm

**DOI:** 10.3389/frobt.2018.00018

**Published:** 2018-03-08

**Authors:** Masayuki Okabe, Seiji Yamada

**Affiliations:** ^1^Faculty of Management and Information Systems, Prefectural University of Hiroshima, Hiroshima, Japan; ^2^Digital Content and Media Sciences Research Divsion, National Institute of Informatics, The Graduate University for Advanced Studies (SOKENDAI), Tokyo, Japan

**Keywords:** constrained clustering, metric learning, boosting, constrained k-means algorithm, kernel matrix learning

## Abstract

This article proposes a constrained clustering algorithm with competitive performance and less computation time to the state-of-the-art methods, which consists of a constrained k-means algorithm enhanced by the boosting principle. Constrained k-means clustering using constraints as background knowledge, although easy to implement and quick, has insufficient performance compared with metric learning-based methods. Since it simply adds a function into the data assignment process of the k-means algorithm to check for constraint violations, it often exploits only a small number of constraints. Metric learning-based methods, which exploit constraints to create a new metric for data similarity, have shown promising results although the methods proposed so far are often slow depending on the amount of data or number of feature dimensions. We present a method that exploits the advantages of the constrained k-means and metric learning approaches. It incorporates a mechanism for accepting constraint priorities and a metric learning framework based on the boosting principle into a constrained k-means algorithm. In the framework, a metric is learned in the form of a kernel matrix that integrates weak cluster hypotheses produced by the constrained k-means algorithm, which works as a weak learner under the boosting principle. Experimental results for 12 data sets from 3 data sources demonstrated that our method has performance competitive to those of state-of-the-art constrained clustering methods for most data sets and that it takes much less computation time. Experimental evaluation demonstrated the effectiveness of controlling the constraint priorities by using the boosting principle and that our constrained k-means algorithm functions correctly as a weak learner of boosting.

## 1 Introduction

Constrained data clustering produces desirable clusters by using two types of pairwise constraints: must-link and cannot-link (Basu et al., 2008). Constraints are a means of supervision that constrain a pair of data points to belong to the same cluster (must-link) or different clusters (cannot-link) or that simply describe whether a pair of data points are similar or dissimilar. In certain applications such as the clustering of faces in videos (Wu et al., 2013) and the assessing of interpatient similarity (Wang et al., 2011) when class labels are not available, constraints are particularly important for enhancing performance.

The constrained clustering algorithms developed so far mainly use these constraints in two ways (Davidson and Basu, 2006). One way is to use them as background knowledge during data partitioning and integration. For example, the COP-k-means constrained k-means algorithm (Wagstaff et al., 2001) uses constraints as knowledge to restrict the data assignment process of the original k-means algorithm. That is, a data point is assigned to a cluster for which the members are constrained to be a must-link with the data point or every member is not constrained as a cannot-link with the data point even if there is another cluster for which the centroid is closer to the data point. Thus, in the COP-k-means algorithm, data are not always assigned to the nearest centroid if the assignment violates a certain constraint. Although the COP-k-means algorithm is quick like the k-means algorithm and completely satisfies the constraints if it only considers must-link constraints, it often fails to satisfy constraints if it has to consider a number of cannot-link constraints.

The other way is to use metric learning, in which a distance measure or a kernel matrix of a data set is learned. Once a metric is learned, it can be used with clustering algorithms instead of a general metric such as the Euclidean distance. In this type of algorithm, a metric is learned as the value of each constrained data pair for the metric comes closer to the preset value of a must-link or cannot-link constraint. For example, if a distance measure is learned in the form of the Mahalanobis distance, a covariance matrix is learned as the value of each constrained data pair. It is small for a must-link constraint and large for a cannot-link one (Davis et al., 2007; Kulis et al., 2009; Liu et al., 2010). With kernel matrix learning, the learned kernel metric is large for must-link and small for cannot-link (Hoi et al., 2007; Li et al., 2008). Although several studies have indicated that metric learning is more effective than the COP-k-means approach, metric learning algorithms can be slow if the number of data points or the data dimension is large (Wu et al., 2009; Jain et al., 2010). The time complexity of metric learning algorithms is often more than *O*(N^2^) in contrast to about *O*(*kN*) for COP-k-means ones, where *N* and *k* are the number of data points and clusters, respectively.

We have developed a constrained clustering algorithm that exploits the computation time advantage of the COP-k-means algorithm and that uses metric learning based on the boosting principle to enhance performance (Dietterich, 2000; Schapire and Freund, 2012). Boosting is a technique for creating a strong hypothesis from an ensemble of weak ones by controlling data priorities. With our algorithm, a hypothesis is a data similarity metric that is represented by a kernel matrix.

We first focused on the fact that the COP-k-means algorithm produces unstable clustering results because the constraints to be satisfied are implicitly decided on the basis of the data assignment order of the k-means process. We modified it so that the constraints are explicitly satisfied in accordance with their priorities. Once the constraint priorities are set, our modified constrained k-means algorithm tries to satisfy the constraints in order of their priorities. We introduced a framework for deciding the priorities on the basis of the boosting principle. This framework controls the constraint priorities in accordance with the boosting principle and makes our constrained k-means algorithm function as a weak learner that iteratively produces weak cluster hypotheses in the form of kernel matrices. These kernel matrices are integrated into a single kernel matrix representing a strong cluster hypothesis that reflects not only pre-given must-link and cannot-link constraints but also latently constrained data pairs.

**Figure 1:**
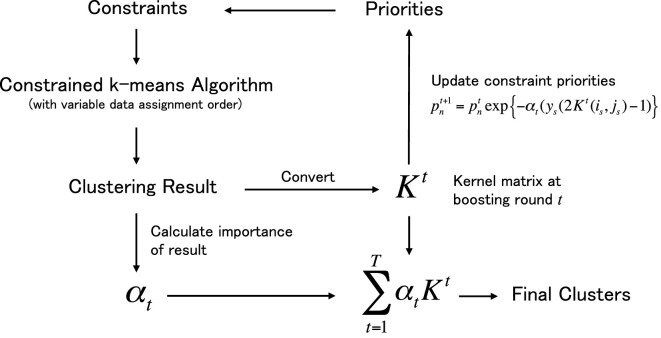
Outline of the proposed metric learning framework under the boosting process. The process starts from the left top and finish in the right bottom.

The proposed metric learning framework is shown in Figure 1. Once given a set of data and constraints (upper left in figure), the framework initiates a boosting process aimed at learning a data similarity metric in the form of kernel matrix *K*. In the boosting process, our constrained k-means algorithm works as a weak learner and produces a weak cluster hypothesis by changing the data assignment order of the k-means process in accordance with the constraint priorities, which are updated in each boosting round *t*. Each cluster hypothesis is converted into a kernel matrix *K^t^*, and the hypotheses are integrated into a single matrix with importance value α*_t_*. The main contributions of our research are as follows.

1. We propose a constrained clustering method with clustering performance competitive to that of state-of-the-art methods and with less computation time. It combines a new constrained k-means algorithm with the boosting principle.
2. We propose a constrained k-means algorithm that considers the priorities of the constraints and functions as a weak learner of boosting and that has computation time competitive to that of the conventional k-means algorithm.

The reminder of this article is structured as follows: Section 2 describes the related work. Section 3 first introduces the COP-k-means algorithm, pointing out that the constraints to be satisfied are randomly decided and then presents our constrained k-means algorithm that considers constraint priorities. Section 4 explains the boosting framework used to control the constraint priorities and describes how our constrained k-means algorithm functions as a weak learner in the framework. Section 5 demonstrates the effectiveness of our algorithm compared with other state-of-the-art constrained clustering algorithms. Section 6 analyzes the experimental results. Finally, Section 7 summarizes the key points and mentions the future work.

## 2 Related Works

The COP-k-means algorithm (Wagstaff et al., 2001) is the first implementation of the constrained k-means method to use pairwise constraints as background knowledge to constrain the k-means data assignment process. Application of the algorithm to the problem of road lane detection from GPS data showed that its performance is dramatically better than that of the conventional k-means algorithm.

Metric learning has been used to exploit pairwise constraints. Basu et al. (2008) proposed a semi-supervised clustering method that combines a constrained k-means approach with a metric learning method that relies on hidden random Markov fields (HMRFs). Davis et al. (2007) proposed a metric learning method based on the information theory. It learns a Mahalanobis metric that distorts the distance between data points on the basis of must-link and cannot-link constraints so as to minimize the relative entropy between multivariate Gaussian distributions parameterized by the initial covariance matrix and the learned matrix. Its performance is better than that of methods using HMRFs. Li et al. (2008) proposed another metric learning approach in which a kernel matrix is learned as a metric that reflects given constraints. They formulated the learning as an optimization problem in which the distance between data points in a high-dimensional space is minimized in accordance with the constraints. Although it needs much computation time since the problem is formulated as a semidefinite programming problem, it outperformed other related methods.

As for the boosting-based metric learning methods, Hertz et al. (2004, 2006) proposed a boosting-based method called DistBoost for learning a distance function. It uses a Gaussian mixture model (GMM) as a weak learner of boosting that learns a hypothesis to be output as a signed confidence measure representing whether a pair of data points originate from the same or different Gaussian sources. This hypothesis is used as a distance function for unlabeled data pairs. Although this approach is similar to ours, its weak learner and the use of constraints are different. Constraints are used for data sampling in the expectation maximization (EM) algorithm to learn GMM parameters. Training a GMM is not easy and generally time consuming, especially when the data dimension is large. Liu et al. (2007) proposed a boosting-based constrained clustering method called BoostCluster. It uses an original boosting framework that creates a feature vector in each round and that can use any type of weak learner. The performance thus depends on the weak learner used. It can be time consuming depending on the data set since it uses eigenvalue decomposition for the square matrix of size *n^2^*, where *n* is the number of data points. Crammer et al. (2002) proposed a boosting-based method for learning a kernel matrix. Although its approach is similar to those of Hertz and ours, the target task is a classification problem that requires labeled data, not constrained data pairs. Thus, it is difficult to use for clustering.

As described above, our algorithm uses a different weak learner for boosting, uses constraints in a unique manner, and is not time consuming compared to other state-of-the-art constrained clustering algorithms.

## 3 Constrained k-Means Algorithm and Data Assignment Order

In this section, we first explain the COP-k-means algorithm and show that it produces unstable clustering results depending on the data assignment order even if the initial k-means cluster centers are fixed. Then we present our modified constrained k-means algorithm that can control the assignment order of constrained data points in accordance with their pre-given priorities. We design it to work as a weak learner of boosting introduced in the next section.

### 3.1 Cop-k-Means Algorithm

The COP-k-means algorithm is based on the k-means algorithm (MacQueen, 1967), which is widely used for various clustering problems because it is easy to implement and quick (Han et al., 2011). The COP-k-means algorithm simply adds a constraint violation checking process to the k-means algorithm. Algorithm 1 shows the COP-k-means procedure (Wagstaff et al., 2001). It satisfies constraints by assigning each data point to the nearest cluster center for which the assignment does not violate a constraint (see line 2 in Algorithm 1). Since this is the only procedural difference from the original k-means algorithm, it is as quick as the original.

**Algorithm 1:** COP-k-means algorithm.

COP-k-means (data set *D*, must-link constraints *Con*_=_ ∈*D* × *D*, cannot-link constraints *Con*_≠_ ∈*D* × *D*)
1: Let *C*_1_,…,*C_k_* be the initial cluster centers.
2: For each point *d_i_* in *D*, assign it to the closest cluster *C_i_* such that VIOLATE-CONSTRAINTS(*d_i_, C_j_, Con*_=_, *Con*_≠_) is false.
3: If no such cluster exists, fail and return.
4: For each cluster *C_i_*, update its center by averaging all of the points *d_i_* that have been assigned to it.
5: Iterate between step 2 and step 4 until convergence.
6: Return *C*_1_, … , *C_k_*.
VIOLATE-CONSTRAINTS(*d, C, Con*_=_, *Con*_≠_)
7: For each (*d, d_m_*) ∈*Con*_=_: If *d_m_* ∉ C, return true.
8: For each (*d, d_c_*) ∈*Con*_=_: If *d_c_* ∈*C*, return true.
9: Otherwise, return false.

However, this algorithm fails and returns nothing if there is no cluster to which it can assign a data point, which can happen when using cannot-link constraints. For example, consider the case of assigning a data point *d_i_* that is constrained by cannot-links (*d_i_, d_c_*) ∈ *Con*_≠_(*c* = 1 ∼ *k*). If every cluster has a data point *d_c_*(*c* = 1 ∼ *k*), there is no cluster to which *d_i_* can be assigned because any assignment would violate a cannot-link constraint. Constrained clustering using cannot-link tends to be an NP-complete problem (Davidson and Ravi, 2005), and it is difficult for the COP-k-means algorithm, which is based on a simple depth-first search without a backtracking mechanism, to solve such a complex problem. One way to overcome this problem is to give up on satisfying all constraints. Since the performance of constrained clustering depends on the constraint set used (Davidson et al., 2006; Davidson, 2012), the constraints to be satisfied should be prioritized if all the constraints cannot be satisfied. Ignoring for the moment the question of which constraints to satisfy, we first modified the COP-k-means algorithm to accept prioritized constraints and then tried to satisfy them on the basis of their priorities.

### 3.2 Constrained k-Means Algorithm with Variable Data Assignment Order

The objectives for modifying the COP-k-means algorithm are summarized as follows.

1. To return a clustering result permitting a partial constraint violation.
2. To enable the constraints to be satisfied in accordance with their given priorities.

Since there are many constrained clustering problems that the COP-k-means algorithm cannot solve, especially when using cannot-link constraints, we formulated our constrained k-means algorithm so that it never aborts even if a constraint violation occurs. We also added a mechanism for satisfying the constraints in order of their pre-given priorities because the constraints to be satisfied should be selected carefully since clustering performance depends on the selection.

The formulated algorithm is shown in Algorithm 2. To ensure that the constraints with higher priorities are satisfied first, we modified the procedure used in the COP-k-means algorithm to assign each data point to a cluster center. In our algorithm, the data pairs related to the constraints are first sorted on the basis of their priorities and then assigned to cluster centers in a descending priority order. Only for the initial assignments is the order randomly decided. Since our algorithm assigns a data pair and not a data point at a time, it has many conditional branches for avoiding constraint violations as much as possible. A data point may be related to more than one constraint, so a data pair may include a data point that has already been assigned in the previous data pair assignment. There are three main branching patterns:

1. Both components of a data pair have not yet been assigned (steps 6–23).
2. One component of a data pair has not yet been assigned (steps 24–35).
3. Both components of a data pair have already been assigned (steps 36–37).

**Algorithm 2:** Constrained k-means algorithm with variable data assignment order.

	INPUT: Data set *D*, must-link constraints with priorities ${\text{Con}}_{=}^{*}$, cannot-link constraints with priorities ${\text{Con}}_{\neq }^{*}$)
	OUTPUT: Clusters *C*_1_, … , *C_k_*
1:	Let *C*_1_, … , *C_k_* be the initial cluster centers.
2:	Let ${\text{Con}}_{\text{all}}^{*}={\text{Con}}_{=}^{*}\cup {\text{Con}}_{\neq }^{*}$.
3:	**While** ${\text{Con}}_{\text{all}}^{*}\neq \emptyset$**do**
4:	Select the constrained data pair (*d_i_, d_j_*) that has the highest constraint priority.
5:	Remove it from ${\text{Con}}_{\text{all}}^{*}$.
6:	**if** Both *d_i_* and *d_j_* are not assigned to cluster centers yet **then**
7:	Let *c_i_* and *c_j_* be the nearest cluster centers for *d_i_* and *d_j_*, respectively.
8:	Let *dist*(*d, c*) be the distance between a data point *d* and a cluster center *c*.
9:	**if** *d_i_* and *d_j_* are constrained as must-link **then**
10:	**if** *dist*(*d_i_, c_i)_* ≤ *dist*(*d_j_, c_j_*) **then**
11:	Assign *d_i_* and *d_j_* to *c_i_*
12:	**else**
13:	Assign *d_i_* and *d_j_* to *c_j_*
14:	**else if** *d_i_* and *d_j_* are constrained as cannot-link **then**
15:	**if** *c_i_* ≠ *c_j_* **then**
16:	Assign *d_i_* and *d_j_* to *c_i_* and *c_j_*, respectively
17:	**else if** *c_i_* = *c_j_* **then**
18:	**if** *dist*(*d_i_, c_i_*) ≤ *dist*(*d_j_, c_j_*) **then**
19:	Assign *d_i_* to *c_i_*.
20:	Assign *d_j_* to the second nearest cluster center after *c_j_*.
21:	**else**
22:	Assign *d_j_* to *c_j_*.
23:	Assign *d_i_* to the second nearest cluster center after *c_i_*.
24:	**else if** *d_i_* is already assigned and *d_j_* is not assigned yet **then**
25:	Let *c_i_* be the cluster center where *d_i_* is assigned
26:	**if** *d_i_* and *d_j_* are constrained as must-link **then**
27:	Assign *d_j_* to *c_i_*
28:	**else if** *d_i_* and *d_j_* are constrained as cannot-link **then**
29:	Assign *d_j_* to the nearest cluster center other than *c_i_*
30:	**else if** *d_i_* is not assigned yet and *d_j_* is already assigned **then**
31:	Let *c_j_* be the cluster center where *d_j_* is assigned
32:	**if** *d_i_* and *d_j_* are constrained as must-link **then**
33:	Assign *d_i_* to *c_j_*
34:	**else if** *d_i_* and *d_j_* are constrained as cannot-link **then**
35:	Assign *d_i_* to the nearest cluster center other than *c_j_*
36:	**else if** both *d_i_* and *d_j_* are already assigned **then**
37:	Do nothing regardless of whether the constraint between *d_i_* and *d_j_* is satisfied.
38:	Assign all unconstrained data points to their nearest cluster centers.
39:	For each cluster *C_i_*, update its center by averaging all of the points *d_i_* that have been assigned to it.
40:	Iterate between step 3 and step 39 until convergence.
41:	Return *C*_1_, … , *C_k_*.

For patterns 1 and 2, more conditional branching is needed depending on whether and how the data pair are constrained (must-link or cannot-link). For pattern 1 and the must-link constraint, both data points are assigned to the cluster center with a distance to the nearest data point less than that of another cluster center (steps 9–13). For pattern 1 and the cannot-link constraint, the data point closest to the nearest cluster center is assigned to that center, and the other data point is assigned to the second nearest cluster center (steps 14–23). For pattern 3, constraint violations are ignored (step 35). The algorithm assigns unconstrained data after the constrained data have been assigned. This assignment procedure is repeated until the cluster set becomes stable.

Our algorithm is based on the assumption that the earlier the assignment of a constrained data pair, the greater the probability of the constraint being satisfied. Although constraint satisfaction is guaranteed only for data pairs with the first or second highest priority,^1^ the constraints with higher priorities should still be easily satisfied because constraint violations tend to occur more frequently as the number of constraints to be considered increases. Since an attempt is made to satisfy constraints with higher priority before the other constraints, there are fewer constraints to be considered. The experimental relationship between constraint priority and the satisfaction rate is discussed in Section 6.

As described in this section, while the COP-k-means algorithm runs fast, it produces unstable clustering results depending on the data assignment order. To complement the drawback, we introduced a modified constrained k-means algorithm that has a mechanism to satisfy constraints in order of their priorities.

## 4 Boosted Constrained k-Means Algorithm

In this section, we introduce a mechanism to automatically decide the data assignment order of our constrained k-means algorithm. It is based on the boosting principle and controls the order appropriately using constraint priority. We first describe why we use boosting and then explain a concrete algorithm that integrates our constrained k-means algorithm into the boosting framework.

The constrained k-means algorithm described in the previous section attempts to satisfy the constraints in accordance with their pre-given priorities. The problem remaining is how to decide the priorities. A higher priority should of course be given to a constraint that is expected to be more effective for clustering. However, it is not easy to estimate the effectiveness. Moreover, even if the effectiveness could be accurately estimated, the number of constraints that can be satisfied in a single run is limited. Given these considerations, we use a boosting technique to enhance the performance of our constrained k-means algorithm. Boosting (Schapiro, 2013) is a method for ensemble learning that produces a better hypothesis from a single weak learner. It enables a weak learner to produce weak hypotheses by adaptively controlling the probability distribution of data occurrence and integrates the hypotheses into a strong hypothesis. Boosting is generally used for classification problems, not for clustering. However, constrained clustering can be viewed as a kind of classification problem in which each data pair is classified into one of two classes (must-link and cannot-link). This means that boosting can be applied to constrained clustering. Since our constrained k-means algorithm can be a weak learner that produces a weak cluster hypothesis, boosting is suitable for our purpose.

Our boosting-based constrained clustering algorithm is shown in Algorithm 3. Its operating principle follows that of AdaBoost (Schapire and Singer, 1999). Unlike the conventional AdaBoost application, our constrained k-means algorithm is used as a weak learner. A weak hypothesis is thus a result of constrained clustering. The priorities of the constraints are assigned and controlled following the conventional AdaBoost procedure since a training data set is a set of constraints in the case of constrained clustering. A weak hypothesis is created in step 3 of our constrained k-means algorithm, which attempts to satisfy the constraints with higher priorities. A cluster hypothesis is represented using a kernel matrix in which each element corresponds to the state of a data pair in the clustering result. The state is represented by 1 or 0, indicating whether the data pair belongs to the same cluster or different clusters. Thus, the kernel matrix is an *N* × *N* semidefinite matrix in which *N* is the number of data points. The proof of semidefiniteness is given in Appendix A.

**Algorithm 3:** Boosted constrained k-means algorithm.

	INPUT: Data set *D*, Constraint set ${\text{Con}}_{\text{all}}^{*}=\left\{\left({\text{i}}_{\text{s}},{\text{j}}_{\text{s}},{\text{y}}_{\text{s}},{\text{p}}_{\text{s}}^{\text{t}}\right)|\text{s}=1∼\text{S}\right\}$
	*i* and *j* are the indexes of a constrained data pair (*d_i_, d_j_*).
	*y* ∈{+1, −1} is the constraint type label.
	*y* takes 1 or −1 if (*d_i_, d_j_*) is constrained as must-link or cannot-link, respectively.
	*p^t^* is the priority of a constrained data pair (*d_i_, d_j_*) at boosting round *t*(*t* = 1 ∼ *T*)
	OUTPUT: Clusters *C*_1_,… , *C_k_*
1:	For each constraint in ${\text{Con}}_{\text{all}}^{*}$, ${\text{p}}_{\text{s}}^{0}\leftarrow \frac{1}{\text{S}}$
2:	**for** *t* = 1 to *T* **do**
3:	Run our constrained k-means algorithm in Algorithm 2 using ${\text{Con}}_{\text{all}}^{*}$.
4:	In accordance with the clustering result, create a kernel matrix *K^t^* as follows.
	${\text{K}}^{\text{t}}\left(\text{a},\text{b}\right)=\left\{\begin{array}{cc} 1 & :\left({\text{d}}_{\text{a}},{\text{d}}_{\text{b}}\right)\;\text{belongs to the same cluster}\\ 0 & :\left({\text{d}}_{\text{a}},{\text{d}}_{\text{b}}\right)\;\text{belongs to a different cluster} \end{array}\right.$
5:	Calculate error rate $\epsilon_{\text{t}}$ using *K^t^*.
	$\epsilon_{\text{t}}=\frac{\frac{1}{2}\sum_{\text{s}=1}^{\text{S}}\;{\text{p}}_{\text{s}}^{\text{t}}\left\{1-{\text{y}}_{\text{s}}\left(2{\text{K}}^{\text{t}}\left({\text{i}}_{\text{s}},\;{\text{j}}_{\text{s}}\right)-1\right)\right\}}{\sum_{\text{s}=1}^{\text{S}}\;{\text{p}}_{\text{s}}^{\text{t}}}$
6:	**if** $\epsilon_{t}=0$ **then**
7:	Let α_*t*_ = α* and go to step 14.
8:	**else if** $\epsilon_{\text{t}}\geq 0.5$ **then**
9:	Let α_*t*_ = 0 and go to step 14.
10:	**else**
11:	Calculate importance α_t_ of *K^t^* using $\epsilon_{\text{t}}$.
	$\alpha_{\text{t}}=\frac{1}{2}\ln\left\{\frac{1-\epsilon_{\text{t}}}{\epsilon_{\text{t}}}\right\}$
12:	Update priority of each constraint ${\text{p}}_{\text{l}}^{\text{t}+1}$.
	${\text{p}}_{\text{s}}^{\text{t}+1}={\text{p}}_{\text{s}}^{\text{t}}\exp\left\{-\alpha_{\text{t}}{\text{y}}_{\text{s}}\left(2{\text{K}}^{\text{t}}\left({\text{i}}_{\text{s}},{\text{j}}_{\text{s}}\right)-1\right)\right\}$
13:	Integrate *K^t^* into *K*.
	$\text{K}=\;\sum_{\text{t}=1}^{\text{T}}\alpha_{\text{t}}{\text{K}}^{\text{t}}$
14:	Run kernel *k*-means using *K* and output clusters *C*.

Once a weak hypothesis is created, it is used to calculate the rate of unsatisfied constraints, i.e., error rate $\epsilon_{t}$ in step 5. Since *y_s_* and *K^t^*(*i_s_, j_s_*) indicate the correct and learned state of a data pair in the clustering result, that is, +1 and −1 indicate whether the data pair components should be in the same cluster or different clusters, respectively, $\epsilon_{t}$ is the sum of the priorities for the unsatisfied constraints. From steps 6 to 12, the importance α*_t_* of kernel matrix *K^t^* is calculated in accordance with the value of $\epsilon_{t}$. There are two exceptional cases depending on the value of $\epsilon_{t}:\epsilon_{t}=0$ means all constraints are satisfied, and $\epsilon_{t}\geq 0.5$ means the weak learner violates a weak learning condition (Schapiro, 2013). Thus, in both cases, the boosting process is stopped, and the final result is created in accordance with each condition. In other cases, the priority of each constraint is updated following step 12. The priorities of the constraints unsatisfied in round *t* of boosting are increased, while the priorities of the satisfied constraints are kept the same. After *T* rounds of the boosting process have been completed, each kernel matrix *K^t^* is integrated into a single matrix *K* in step 13. This matrix is also semidefinite (see Appendix A). We can use the kernel k-means algorithm (Girolami, 2002) or other kernel-based clustering algorithms with *K* to obtain the final clustering result.

We use our constrained k-means algorithm as the weak learner for boosting. The probability distribution of the constraints is used to set the data assignment order. In general, boosting can be interpreted as an optimization process for finding a hypothesis that minimizes a loss function. In the case of boosting, the loss function is

$$\Sigma_{s=1}^{S}\exp\left(-y_{s}K\left(i_{s},j_{s}\right)\right),$$

where kernel *K* minimizes the function for use as an optimal hypothesis. Although the function considers only constrained data pairs, unconstrained pairs are also involved in the learning process. This boosting process can thus be viewed as transductive learning in which prediction is executed in conjunction with learning.

The boosting process introduced in this section is an approach to enhance the performance of our constrained k-means algorithm. Since it is difficult for the constrained k-means algorithm to satisfy all constraints by itself, we use an ensemble approach that tries to satisfy as many constraints as possible by majority vote of diverse clustering results. The boosting process produces the diversity by controlling the constraint priority to decide the data assignment order of the constrained k-means algorithm. While kernel matrix is a representation of a clustering result, it is suitable to represent the aggregation of data pair relationships.

## 5 Evaluation

In this section, we evaluate the performance of our method by comparing it with other state-of-the-art methods on various data sets. We first describe about details of experiments such as data sets, the evaluation metric, methods to be compared, and other settings. We then show the clustering performance and the computation time of each method.

We used 12 data sets from 3 data sources (see Table 1). We used six data sets from the UCI repository,^2^ a well-known data source for supervised and unsupervised learning, that had different numbers of data points, classes, and attributes. We used three from CLUTO,^3^ a data source for evaluating clustering algorithms with high-dimensional text data sets. We used three from Shape,^4^ a data source providing data sets of two-dimensionally scattered data consisting of characteristically shaped clusters. Since the data sets from CLUTO were text data, we transformed each data item into a unit feature vector by using the term frequency–inverse document frequency (tf-idf) method (Salton and McGill, 1983).

**Table 1:** Data sets.

Data set	UCI					
	Iris	Ecoli	Wdbc	Sonar	Glass	Libras
No. of data points	150	336	569	208	214	360
No. of clusters	3	7	2	2	6	15
No. of attributes	4	8	30	60	9	90

**Data set**	**CLUTO**			**Shape**		
	**Tr11**	**Tr12**	**Tr23**	**Flame**	**Pathbased**	**Spiral**

No. of data points	414	313	204	240	300	312
No. of clusters	9	8	6	2	3	3
No. of attributes	6,429	5,804	5,832	2	2	2

Each data set had a set of class labels, and we assumed that a group of data with the same class label was a cluster. We used the normalized mutual information (NMI) metric (Strehl and Ghosh, 2003) to evaluate the clustering results. Let *N* and *k* be the number of data points and clusters, *C* be the set of produced clusters, and *T* be the set of correct clusters.

$$\text{NMI}=\frac{\sum_{i=1}^{k}\;\sum_{j=1}^{k}\;n_{i,j}^{C,T}\log\left(\frac{N\cdot n_{i,j}^{C,T}}{n_{i}^{C}n_{j}^{T}}\right)}{\sqrt{\left(\sum_{i=1}^{k}\;n_{i}^{C}\log\frac{n_{i}^{C}}{N}\right)\left(\sum_{j=1}^{k}\;n_{j}^{T}\log\frac{n_{j}^{T}}{N}\right)}},$$

where $n_{i}^{C}$ is the number of data points belonging to the *i*th cluster in *C*, $n_{j}^{T}$ is the number of data points belonging to the *j*th cluster in *T*, and $n_{i,j}^{C,T}$ is the number of data points belonging to both the *i*th cluster in *C* and the *j*th cluster in *T*. NMI represents the consistency between *C* and *T*, giving a value between 0 and 1. The clustering result is assumed to be better if the value is larger.

### 5.1 Methods

We compared our boosted constrained k-means (BCKM) algorithm^5^ with seven algorithms. For our algorithm, we set the number of rounds of boosting to 100, *ϵ_t_* = 0, and α* = 100 for all data sets.

- **KBST** This algorithm is an alternative version of the DistBoost algorithm (Hertz et al., 2004) and is referred to as KernelBoost (Hertz et al., 2006). It creates a kernel matrix from pairwise constraints. The source code^6^ was provided by the authors. We first created a kernel matrix using this algorithm and then used the kernel k-means algorithm with the kernel matrix to create the final clustering results. We set the number of models for GMM to the number of clusters for each data set. We used the default values for the other parameters. We set the number of rounds of boosting to 100.
- **BSTC** This is the BoostCluster algorithm (Liu et al., 2007). We used the k-means algorithm for the basic clustering and the kernel k-means algorithm with the kernel matrix created by BoostCluster to create the final clustering results. We set the number of dimensions for new feature vectors that the BoostCluster algorithm created in each boosting round to the same number of dimensions for the original feature vectors. We again set the number of rounds of boosting to 100.
- **ITML** This algorithm is called the information theoretic metric learning algorithm (Davis et al., 2007), a state-of-the-art distance learning algorithm. The source code^7^ was provided by the authors. We used the k-means algorithm with a distance matrix created by ITML to create the final clustering results. We used the default values for the parameters of ITML.
- **PCP** This is a state-of-the-art algorithm for kernel matrix learning that is based on the semidefinite programming (Li et al., 2008). We used the kernel k-means algorithm to create the final clustering results. For the semidefinite programming solver, we used SDPT3^8^ for which the parameters were set to the default values.
- **SPCL** This is a spectral clustering algorithm proposed by Kamvar et al. (2003). We set the number of dimensions for new feature vectors created after Eigen decomposition to the same number of dimensions for the original feature vectors.
- **CKM** This algorithm uses the constrained k-means algorithm described in Section 3 as a standalone algorithm. We used it to evaluate the effectiveness of ensemble learning. We randomly set the data assignment order.
- **RCKM** This is an alternative of our proposed algorithm in which the data assignment order is randomly set. We used it to evaluate the effectiveness of using boosting to control the data assignment order. We set the number of rounds of boosting to 100.

We used the k-means++ algorithm (Arthur and Vassilvitskii, 2007) to set the initial cluster centers in the k-means algorithm. For the Shape data sets, we used the kernel k-means algorithm and the radial basis function (RBF) kernel with local scaling (Zelnik-Manor and Perona, 2005). We created an initial affinity matrix for the PCP and SPCL algorithms by using a linear kernel for the UCI and CLUTO data sets and an RBF kernel with local scaling for the Shape data sets. For the RBF kernel, we set the number of the *n*th neighbor to 7. Each algorithm was implemented as a MATLAB program and executed on the same PC (CPU Core i7, 3.40 GHz, 16 GB memory).

### 5.2 Other Settings

The constraints were first created by randomly selecting a pair of data points and assigning to it a must-link or cannot-link label in accordance with whether the pair components had the same or different class labels. For each data set, testing was done using three different numbers of constraints: 1, 5, and 10% of the total number of data pairs. That is, if the number of data points was 150 and the percentage was 1, we created 111($≒0.01{*}_{150}{\text{C}}_{2}$) constraints. In addition, we created 10 different sets of constraints for each percentage because clustering performance suffers from ill combinations of constraints. For our k-means algorithm, we created 10 different sets of initial cluster centers for each data set. One hundred trials were conducted for each algorithm and percentage of constraints.

### 5.3 Results

#### 5.3.1 Clustering Performance

The clustering performance (i.e., the NMI metric) for each method is summarized in Table 2, with the best performance shown in bold. We provide the average and the SD of nmi values, each of which was calculated for a clustering result with a certain set of constraints. Note that results could not be obtained for some combinations of methods and data sets. KBST could not produce results for the Sonar, Glass, and Libras data sets because the program crashed during the creation of the GMM models. We eliminated the results for KBST, BSTC, and ITML for the CLUTO and Shape data sets because the results were significantly worse than for the other data sets. We also eliminated the results for SPCL for all UCI and CLUTO data sets for the same reason. Thus, the algorithms compared with our proposed BCKM algorithm for all data sets were PCP, CKM, and RCKM.

**Table 2:** Clustering performance (NMI metric).

Method	Iris (UCI)			Ecoli (UCI)		
	1%	5%	10%	1%	5%	10%
BCKM	0.81 ± 0.08	**0.99 **± 0.01	**0.99 **± 0.00	**0.66 **± 0.03	**0.81 **± 0.05	**0.81 **± 0.05
KBST	0.86 ± 0.04	0.89 ± 0.01	0.91 ± 0.04	0.63 ± 0.01	0.65 ± 0.02	0.67 ± 0.01
BSTC	0.75 ± 0.10	0.75 ± 0.07	0.70 ± 0.08	0.49 ± 0.03	0.49 ± 0.03	0.48 ± 0.04
ITML	**0.91 **± 0.02	0.92 ± 0.01	0.92 ± 0.01	0.57 ± 0.02	0.59 ± 0.02	0.59 ± 0.02
PCP	0.71 ± 0.03	0.98 ± 0.01	**0.99 **± 0.03	0.54 ± 0.02	0.65 ± 0.02	0.77 ± 0.03
CKM	0.78 ± 0.04	0.75 ± 0.02	0.75 ± 0.01	0.60 ± 0.03	0.62 ± 0.06	0.63 ± 0.08
RCKM	0.81 ± 0.03	0.88 ± 0.03	0.88 ± 0.03	0.62 ± 0.02	0.61 ± 0.01	0.61 ± 0.01

**Method**	**Wdbc (UCI)**			**Sonar (UCI)**		
	**1%**	**5%**	**10%**	**1%**	**5%**	**10%**

BCKM	**0.90 **± 0.07	0.96 ± 0.03	0.99 ± 0.00	**0.37 **± 0.23	0.97 ± 0.03	0.99 ± 0.00
KBST	0.80 ± 0.00	0.80 ± 0.00	0.85 ± 0.02	–	–	–
BSTC	0.43 ± 0.03	0.43 ± 0.03	0.45 ± 0.02	0.03 ± 0.03	0.09 ± 0.04	0.11 ± 0.03
ITML	0.49 ± 0.01	0.61 ± 0.02	0.63 ± 0.02	0.20 ± 0.05	0.36 ± 0.04	0.38 ± 0.05
PCP	**0.90 **± 0.02	**1.00 **± 0.00	**1.00 **± 0.00	0.03 ± 0.02	**0.99 **± 0.01	**1.00 **± 0.00
CKM	0.42 ± 0.24	0.62 ± 0.23	0.73 ± 0.30	0.05 ± 0.05	0.18 ± 0.20	0.51 ± 0.30
RCKM	0.89 ± 0.04	0.96 ± 0.02	0.98 ± 0.01	0.04 ± 0.04	0.27 ± 0.17	0.47 ± 0.22

**Method**	**Glass (UCI)**			**Libras (UCI)**		
	**1%**	**5%**	**10%**	**1%**	**5%**	**10%**

BCKM	**0.36 **± 0.04	**0.73 **± 0.05	**0.82 **± 0.05	0.54 ± 0.02	**0.71 **± 0.02	**0.84 **± 0.02
BSTC	0.28 ± 0.04	0.30 ± 0.04	0.31 ± 0.04	0.51 ± 0.05	0.56 ± 0.02	0.57 ± 0.02
ITML	0.35 ± 0.02	0.34 ± 0.01	0.30 ± 0.04	**0.61 **± 0.03	0.63 ± 0.03	0.60 ± 0.03
PCP	0.30 ± 0.02	0.56 ± 0.04	0.69 ± 0.04	0.32 ± 0.02	0.17 ± 0.01	0.21 ± 0.01
CKM	0.35 ± 0.02	0.46 ± 0.10	0.54 ± 0.11	0.56 ± 0.01	0.57 ± 0.01	0.58 ± 0.01
RCKM	0.35 ± 0.03	0.36 ± 0.02	0.36 ± 0.02	0.57 ± 0.01	0.57 ± 0.01	0.57 ± 0.01

**Method**	**Tr11 (CLUTO)**			**Tr12 (CLUTO)**		
	**1%**	**5%**	**10%**	**1%**	**5%**	**10%**

BCKM	**0.70 **± 0.03	**0.86 **± 0.03	**0.87 **± 0.04	0.68 ± 0.06	**0.90 **± 0.03	**0.95 **± 0.03
PCP	0.66 ± 0.02	0.58 ± 0.03	0.75 ± 0.02	**0.72 **± 0.04	0.44 ± 0.03	0.68 ± 0.05
CKM	0.05 ± 0.05	0.18 ± 0.20	0.51 ± 0.30	0.60 ± 0.03	0.62 ± 0.06	0.63 ± 0.08
RCKM	0.64 ± 0.03	0.64 ± 0.03	0.64 ± 0.03	0.64 ± 0.06	0.64 ± 0.07	0.64 ± 0.07

**Method**	**Tr23 (CLUTO)**			**Flame (Shape)**		
	**1%**	**5%**	**10%**	**1%**	**5%**	**10%**

BCKM	0.38 ± 0.05	**0.75 **± 0.07	**0.83 **± 0.07	0.98** **± 0.03	**1.00 **± 0.00	**1.00 **± 0.00
PCP	0.42** **± 0.03	0.57** **± 0.04	0.69** **± 0.06	**0.99 **± 0.01	**1.00 **± 0.00	**1.00 **± 0.00
SPCL	–	–	–	0.92** **± 0.01	0.97** **± 0.02	0.99** **± 0.01
CKM	**0.56 **± 0.01	0.57** **± 0.01	0.58** **± 0.01	0.78** **± 0.38	0.91** **± 0.28	0.80** **± 0.39
RCKM	0.37** **± 0.04	0.39** **± 0.04	0.39** **± 0.03	0.98** **± 0.02	**1.00 **± 0.00	**1.00 **± 0.00

**Method**	**Pathbased (Shape)**			**Spiral (Shape)**		
	**1%**	**5%**	**10%**	**1%**	**5%**	**10%**

BCKM	0.85** **± 0.14	0.87** **± 0.14	0.91** **± 0.13	**0.96 **± 0.09	0.93** **± 0.11	0.95** **± 0.10
PCP	**0.95 **± 0.07	**0.97 **± 0.09	**0.97 **± 0.08	0.88** **± 0.13	0.97** **± 0.08	**0.99 **± 0.05
SPCL	0.86** **± 0.07	0.93** **± 0.03	**0.97 **± 0.02	0.64** **± 0.14	**0.98 **± 0.05	**0.99 **± 0.03
CKM	0.46** **± 0.22	0.74** **± 0.07	0.76** **± 0.04	0.17** **± 0.13	0.58** **± 0.00	0.58** **± 0.00
RCKM	0.72** **± 0.21	0.91** **± 0.15	0.93** **± 0.12	0.05** **± 0.05	0.07** **± 0.07	0.09** **± 0.09

The BCKM algorithm had the best results in most cases, particularly for 6 of the 12 data sets when the constraint percentage was 1(hereafter “6/12(1%)”). It similarly had the best results for 5 and 10%: 8/12(5%) and 8/12(10%). Even when it missed the top rank, its results were competitive in most cases. The second best method was PCP: 4/12(1%), 4/12(5%), and 6/12(10%). The performance of BCKM stably improved with an increase in the number of constraints, especially for the Sonar, Glass, Libras, Tr11, Tr12, and Tr23 data sets. In addition, only BCKM showed improvement for the Libras and Tr12 data sets, whereas the others showed no gain or even a reduction. Compared with CKM and RCKM, BCKM was significantly better for the Sonar and Spiral data sets.

#### 5.3.2 Computation Time

The average computation times for one trial for each algorithm are summarized in Table 3. The computation time for the BCKM algorithm was proportional to the number of constraints. BCKM was much faster than KBST. Although BSTC and ITML were faster or competitive for the UCI data sets, for which the number of attributes was relatively small, their computation times were significantly higher for the CLUTO data sets, which had a large number of attributes. BCKM was faster than PCP for most data sets, especially for the Wdbc and Tr11 data sets, in which the number of data points was relatively large.

**Table 3:** Computation time (s).

Method	Iris (UCI)			Ecoli (UCI)		
	1%	5%	10%	1%	5%	10%
BCKM	0.02	0.27	0.49	1.72	14.71	13.96
KBST	134.71	172.94	122.15	469.88	337.49	232.19
BSTC	0.68	1.22	1.73	3.95	5.00	6.08
ITML	0.23	2.01	2.21	4.41	4.47	4.50
PCP	0.59	1.38	2.45	3.68	28.62	131.93
CKM	0.01	0.01	0.02	0.02	0.09	0.24

**Method**	**Wdbc (UCI)**			**Sonar (UCI)**		
	**1%**	**5%**	**10%**	**1%**	**5%**	**10%**

BCKM	13.35	47.52	30.60	1.30	4.41	6.12
KBST	551.49	117.36	39.36	–	–	–
BSTC	4.31	6.19	8.51	1.68	1.94	2.24
ITML	3.56	3.86	4.18	1.02	7.45	8.67
PCP	24.92	354.22	1870.17	1.44	3.85	14.15
CKM	0.04	0.06	0.09	0.01	0.01	0.01

**Method**	**Glass (UCI)**			**Libras (UCI)**		
	**1%**	**5%**	**10%**	**1%**	**5%**	**10%**

BCKM	1.29	4.51	7.47	1.04	13.26	48.36
BSTC	3.11	5.02	6.74	4.98	5.48	6.40
ITML	1.41	4.09	4.33	25.34	30.56	33.96
PCP	1.19	7.02	23.71	5.69	67.90	147.37
CKM	0.01	0.02	0.03	0.04	0.08	0.11

**Method**	**Tr11 (CLUTO)**			**Tr12 (CLUTO)**		
	**1%**	**5%**	**10%**	**1%**	**5%**	**10%**

BCKM	8.21	21.86	23.00	3.68	13.51	20.85
PCP	9.47	103.37	268.57	3.41	23.79	80.62
CKM	0.12	0.15	0.20	0.08	0.09	0.11

	**Tr23 (CLUTO)**			**Flame (Shape)**		
**Method**	**1%**	**5%**	**10%**	**1%**	**5%**	**10%**

BCKM	0.81	4.75	6.31	0.03	0.07	0.17
PCP	1.70	4.51	22.94	1.34	3.94	16.00
SPCL	–	–	–	0.12	0.13	0.11
CKM	0.03	0.03	0.04	0.02	0.03	0.02

**Method**	**Pathbased (Shape)**			**Spiral (Shape)**		
	**1%**	**5%**	**10%**	**1%**	**5%**	**10%**

BCKM	0.26	0.65	2.01	0.91	1.00	2.15
PCP	2.64	11.52	80.48	2.27	12.99	83.55
SPCL	0.15	0.15	0.16	0.16	0.16	0.17
CKM	0.02	0.05	0.05	0.04	0.02	0.03

In summary, experimental results shows that our method has competitive clustering performance relative to the other state-of-the-art ones and its computation time is proportional to the number of constraints. We further discuss about the property of our method in the next section.

## 6 Discussions

In this section, we analyze the behavior of our method in more detail. We first verify the effectiveness of data assignment order mechanism of our method by showing the relationship between the number of boosting rounds and the clustering performance and between data assignment order and constraint satisfaction rate. We then discuss the property of our method from both the performance and computation time points of views. We finally compare the behavior of our method with others by visualizing clustering results.

### 6.1 Effectiveness of Data Assignment Order

As shown by our evaluation, the BCKM algorithm outperformed the CKM and RCKM algorithms for most of the data sets. RCKM is a kind of bagging method (Breiman, 1996) in which CKM is a weak learner. Since it outperformed or showed competitive performance against CKM for 10 of the 12 data sets, we can conclude that the data assignment order of the constrained k-means algorithm affects clustering performance. While there may be some assignment orders that are better in terms of creating clusters, the boosting-based data assignment method of BCKM is a promising way to enhance the performance of the constrained k-means algorithm as the results showed that BCKM significantly outperformed RCKM for 9 of the 12 data sets. We thus focus the rest of the discussion in this section on BCKM.

The BCKM algorithm can cope with exceptional cases such as when a weak learner satisfies all the constraints or fails to satisfy more than half the constraints, as shown in steps 6–9 of Algorithm 3. We identified instances of the former case in our evaluation, where BCKM terminated the boosting process before the number of rounds reached the preset value of *T* (100). Table 4 shows the average number of boosting rounds when BCKM terminated the process. The 1, 5, and 10% again indicate the percentage of constraints for all data pairs. The average number was less than 100 in many cases. For some data sets, such as Iris, Flame, Pathbased, and Spiral, the number was less than around 20. For those data sets, BCKM showed high performance (NMI value close to 1). Interestingly, the average number for 5% was less than the value for 10% for half the data sets. Although it would be reasonable to think that BCKM needs more boosting rounds as the number of constraints increases, this is not necessarily true depending on the task.

**Table 4:** Number of rounds before boosting was terminated for BCKM algorithm.

	Iris	Ecoli	Wdbc	Sonar	Glass	Libras
1%	3.57	54.66	99.07	92.57	73.21	22.47
5%	13.85	65.86	83.17	98.79	100.00	100.00
10%	13.82	34.06	26.50	73.00	92.13	100.00

	**Tr11**	**Tr12**	**Tr23**	**Flame**	**Pathbased**	**Spiral**

1%	61.75	46.01	29.26	1.23	6.40	20.26
5%	92.26	100.00	99.30	1.09	5.86	8.15
10%	59.16	99.37	70.80	1.39	9.95	9.80

**Figure 2:**
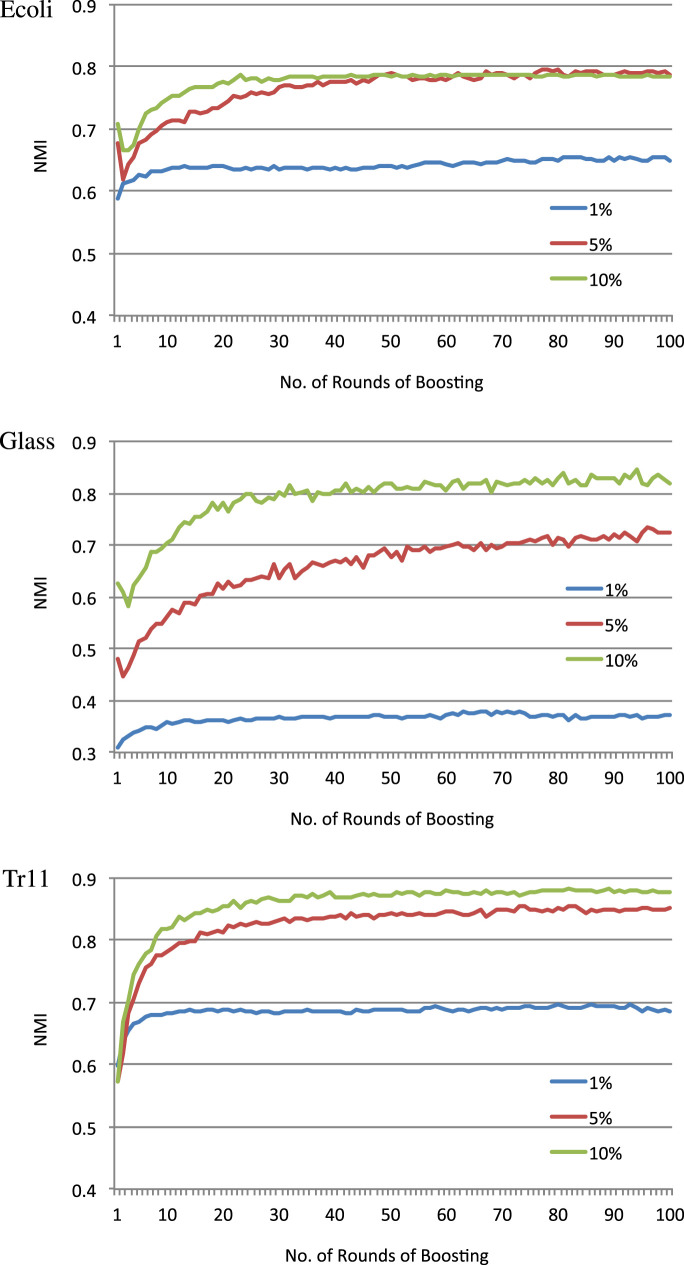
NMI at each round of boosting.

Since BCKM needs less computation time due to fewer boosting rounds, we can adjust parameter *T* to optimize the trade-off between an acceptable computation time and the performance necessary for the target task. Figure 2 shows typical relationships between the number of boosting rounds and NMI for three data sets Ecoli, Glass, and Tr11. The graphs plot the NMI values calculated by tentatively obtaining a kernel matrix *K* (step 13 in Algorithm 3) and using it to execute kernel k-means clustering at each boosting round *t*. In most cases, NMI sharply increased by the 30th boosting round, while in some cases, such as 5 and 10% for Ecoli and Glass, NMI temporarily dropped for a few rounds before increasing again. This illustrates the advantage of ensemble learning in which a few bad weak hypotheses do not significantly affect the overall performance. Unlike CKM, BCKM does not depend on a single randomly set data assignment order that may or may not work and can recover even if the initial data assignment order does not work.

**Figure 3:**
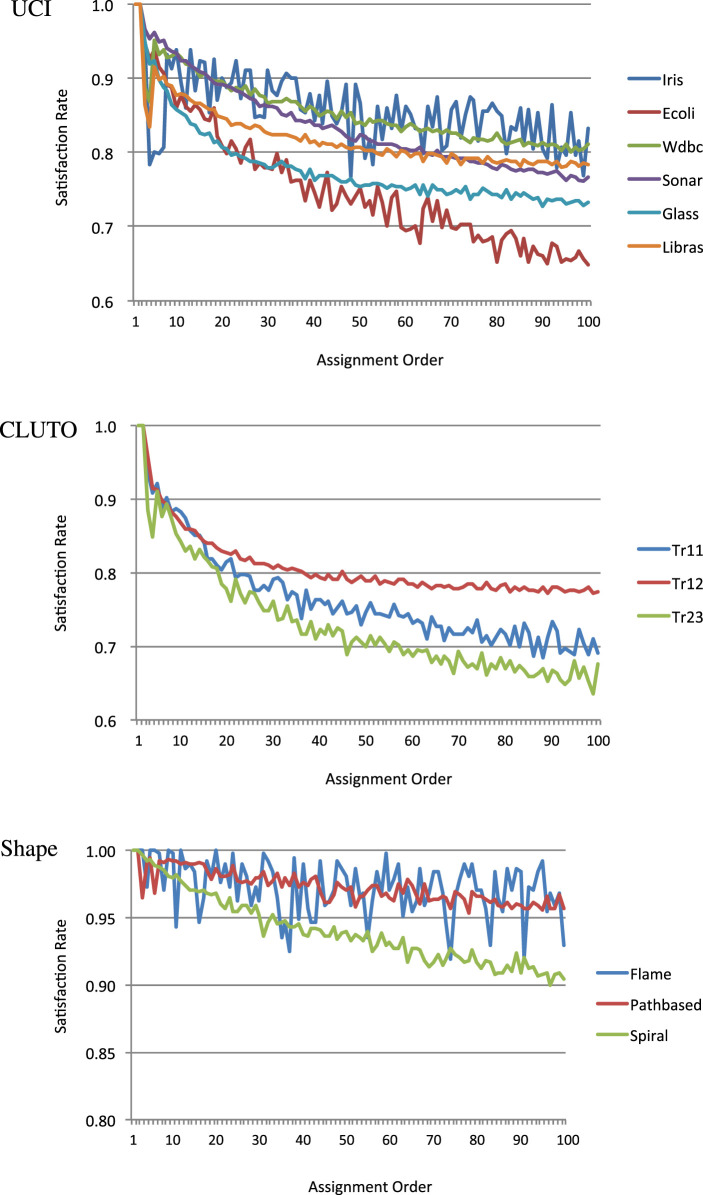
Rate of satisfied constraints with *n*th priority.

As described in the last paragraph of Section 3, our algorithm is based on the assumption that the earlier the assignment of a constrained data pair, the greater the probability of the constraint being satisfied. Proofs that the first and second constraints in the data assignment order are guaranteed to be satisfied are given in Appendix 2. Here, we consider how many of the subsequent order constraints are satisfied. The relationships between the data assignment order and the satisfaction rates for all data sets are plotted in Figure 3. The rates for the constraints with first or second data assignment orders were 100% in all cases. The rates for the other constraints gradually decreased with the assignment order for most data sets. While some data sets such as Iris and Flame showed unstable patterns, our assumption was valid for most data sets. Thus, the BCKM algorithm should work well for most data sets.

### 6.2 Computation Time

Finally, we compare the BCKM and other algorithms from both the performance and the computation time points of view. Although BCKM is based on an approach similar to that of KBST, it was better from both points of view. Since the boosting frameworks used in both methods are quite similar, the advantage must be due to the quality of our original weak learner. From the performance point of view, our weak learner, a constrained k-means method with variable data assignment order, utilizes not only must-link but also cannot-link constraints, while the KBST weak learner, a constrained GMM, basically considers only must-link constraints. From the computation time point of view, our weak learner is much quicker than the KBST one since the EM algorithm used to estimate the GMM parameters generally needs more calculation time compared with that of the k-means algorithm.

BCKM showed better performance than BSTC, which is also based on the boosting framework. Since BSTC creates a new feature vector in each boosting round by Eigen decomposition, it is not suitable for data sets with a small or sparse feature space. Furthermore, it needs much computation time for data sets with a large feature space. Similarly, ITML is not suitable for data sets with a sparse feature space and needs much computation time if the feature space is large because ITML needs to learn a Mahalanobis distance matrix that has *F* × *F* elements, where *F* is the number of features. SPCL is also not suitable for data sets that have a sparse feature space and needs much computation time if the feature space is large because it also uses Eigen decomposition.

PCP worked well for all data sets and showed performance competitive to that of BCKM for some data sets. However, it needs a semidefinite programming solver, which requires more computation time than BCKM if the number of data sets is large.

In summary, our method is well balanced in terms of both performance and computation time since it achieves competitive performance with less computation time against other methods for many data sets. However, it needs a certain number of constraints because, if only a small number of constraints are given, the weak learner can satisfy all the constraints in the first boosting round, which results in the same performance as that of CKM. Thus, the number of constraints should exceed the number that CKM can satisfy all by itself.

### 6.3 Visualization

**Figure 4:**
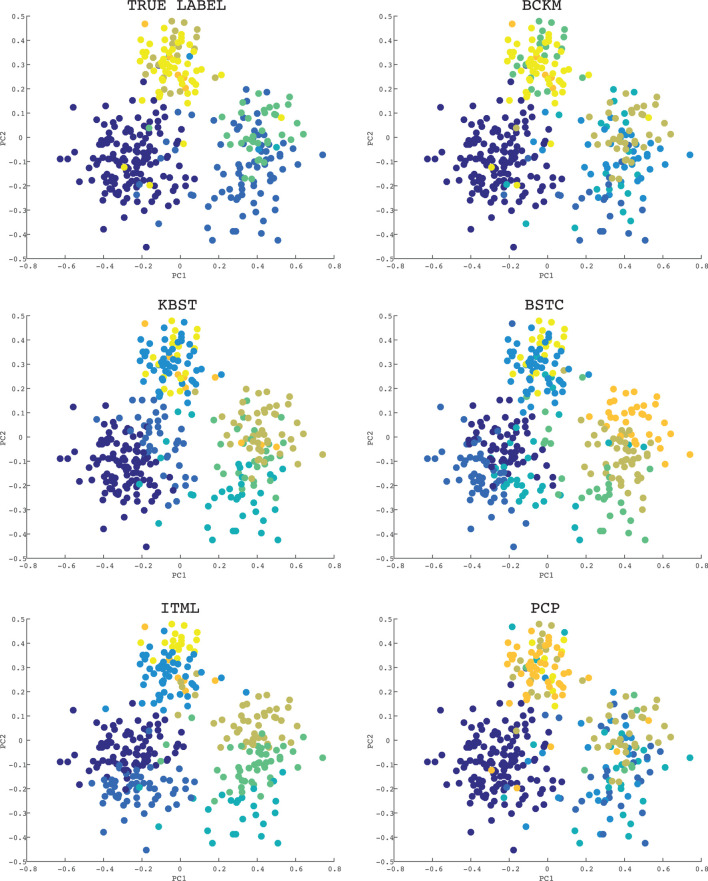
Visualization of clustering results for the Ecoli data set: the constraint ratio is 10%. The title of each graph is the name of method. The graph with title “TRUE LABEL” shows the correct clustering result. The color of each point shows the cluster group.

Finally, we visualize the clustering result of each method on a data set. Figure 4 shows the results on the Ecoli data set where the constraint ratio is 10%. Since the dimension is more than two, we used the principle component analysis (PCA) to visualize (Nguyen et al., 2008). Axes PC1 and PC2 in each graph corresponds to the first and the second component, respectively. The graph with title “TRUE LABEL” shows the correct clustering results. The color of each point shows the cluster group. The Ecoli data set has 8 clusters. As shown in the graphs, BCKM and PCP are similar to the correct result, while other methods even failed to make the largest cluster (left lower cluster with purple color).

In this section, we first showed that the clustering performance of our method increases according to the number of boosting rounds, and we can choose an appropriate number depending on the required performance. We then experimentally verified the assumption that the earlier the assignment of a constrained data pair, the greater the probability of the constraint being satisfied. This is the reason why our method works well. We also discussed that our method has well-balanced properties in terms of clustering performance and computation time. We finally compared the actual behavior of each method by visualizing clustering results on a data set.

## 7 Conclusion

Our proposed constrained clustering algorithm balances the performance and computation time. We focused on the computation time advantage of the COP-k-means algorithm and improved its performance by incorporating a mechanism for accepting constraint priorities and a framework of kernel matrix learning that is based on the boosting principle. In this framework, our constrained k-means algorithm works as a weak learner that iteratively produces a weak hypothesis in the form of a kernel matrix by changing the data assignment order of the k-means process, which is set on the basis of constraint priorities controlled by the boosting principle.

Evaluation results showed that our method is better or competitive to other state-of-the-art methods in terms of clustering performance and computation time. They also showed that the number of boosting rounds can be adjusted to optimize the trade-off between clustering performance and computation time and that our constrained k-means algorithm correctly works as a weak learner of the boosting to satisfy constraints in accordance with their priorities. Our algorithm works well regardless of the fraction of must-link and cannot-link constraints, while it needs a certain number of constraints to bring out the strength of boosting.

Boosting is an ensemble learning approach. Although we tested a bagging approach, another promising approach is random forests. Its use remains for future work.

## Author Contributions

MO and SY contributed to making the idea of the research, implementing the proposed method and other ones to be compared, analyzing the results of the experiments, writing the paper, and final approval of the version to be published.

## Conflict of Interest Statement

The authors declare that the research was conducted in the absence of any commercial or financial relationships that could be construed as a potential conflict of interest.

## Footnotes

^1^The proof is in Appendix B.

^2^http://archive.ics.uci.edu/ml/.

^3^http://glaros.dtc.umn.edu/gkhome/cluto/cluto/download.

^4^http://cs.joensuu.fi/sipu/datasets/.

^5^The source code is available at https://github.com/mokabe1567/bckm.

^6^http://www.scharp.org/thertz/code.html.

^7^http://www.cs.utexas.edu/~pjain/itml/.

^8^http://www.math.nus.edu.sg/~mattohkc/sdpt3.html.

## Appendix

### A Proof That *K^t^* and *K* Are Semidefinite

To prove that *K****_t_*** is semidefinite, we have to show that *xK_t_x′* ≥ 0 *x′* is the transposed matrix of *x* for any vector *x* = {*x*_1_, ⋯  , *x_N_*}.

Suppose we obtain a set of *k* clusters at step 5 in Algorithm 4 and let $d_{i}^{k}\left(i=1,\cdots \;,n_{k}\right)$ be data that belongs to the *k*th cluster.

If we replace rows (or columns) of *K^t^* with data in the same cluster to serially align as follows:

$$\underset{\mathrm{cluster}1}{\underset{︸}{d_{1}^{1},\cdots ,d_{n_{1}}^{1}}},\underset{\mathrm{cluster}2}{\underset{︸}{d_{2}^{1},\cdots ,d_{n_{2}}^{2}}},\cdots \underset{\mathrm{clusterk}}{\underset{︸}{d_{k}^{1},\cdots ,d_{n_{k}}^{k}}}$$

where *n_k_* is the number of data points in the *k*th cluster, we can represent *K^t^* as

$$K_{t}=\left(\begin{array}{cccc} A_{1} & O & O & O\\ O & A_{2} & O & O\\ O & O & \ddots & O\\ O & O & O & A_{k} \end{array}\right),$$

where *A_k_* is an *n_k_* × *n_k_* matrix for which the elements are all 1 and *O* is a zero matrix. Then we can represent *xk_t_x′* as follows:

$$\begin{array}{ll} xK_{t}x^{\prime } & =\\ & =xA_{1}x^{\prime }+xA_{2}x^{\prime }+\cdots +xA_{k}x^{\prime }\\ & ={\left\{\sum_{i=1}^{n_{1}}\;x_{i}\right\}}^{2}+{\left\{\sum_{i=n_{1}+1}^{n_{1}+n_{2}}\;x_{i}\right\}}^{2}+\cdots \\ & \;+{\left\{\sum_{i=N-n_{k}+1}^{N}\;x_{i}\right\}}^{2}\\ & \;\geq 0. \end{array}$$

Thus *K^t^* is semidefinite.

Furthermore, for *K*, we can transform

$$\begin{array}{ll} {\mathrm{xKx}}^{\prime } & =x\left\{\sum_{t=1}^{T}\;\alpha_{t}K_{t}\right\}x^{\prime }\\ & =\sum_{t=1}^{T}\;\alpha_{t}xK_{t}x^{\prime }, \end{array}$$

where α*_t_* > 0, and *xK_t_x′* ≥ 0, meaning that *xKx′* ≥ 0. Thus, *K* is also semidefinite.

### B Proof That Constraints with First or Second Priority Are Guaranteed to Be Satisfied

We assume that the constraints are consistent each other and guaranteed to be satisfied theoretically.

#### B.1 Constraint with First Priority

This constraint is guaranteed to be satisfied since there are no other constraints when it is being satisfied.

#### B.2 Constraint with Second Priority

Let a constrained data pair be assigned with the first order (*d*_1_, *d*_2_).

There are two patterns for a combination of constrained data pairs to be assigned with the second order.

- If the first and second pairs have the same data points, let the second pair be (*d*_1_, *d*_3_) or (*d*_2_, *d*_3_).
- If the first and second pairs do not have the same data points, let the second pair be (*d*_3_, *d*_4_).

For each case, we show how to satisfy the constraint.

- For (*d*_1_, *d*_3_) or (*d*_2_, *d*_3_)We here describe the case of (*d*_1_, *d*_3_).
  - If (*d*_1_, *d*_2_) and (*d*_1_, *d*_3_) are both must-link pairs, we can satisfy the must-link constraint of (*d*_1_, *d*_3_) by assigning *d*_3_ to the same cluster as *d*_1_ and *d*_2_.
  - If (*d*_1_, *d*_2_) is a must-link pair and (*d*_1_, *d*_3_) is a cannot-link pair, we can satisfy the cannot-link constraint of (*d*_1_, *d*_3_) by assigning *d*_3_ to a different cluster than *d*_1_ and *d*_2_.
  - If (*d*_1_, *d*_2_) is a cannot-link pair and (*d*_1_, *d*_3_) is a must-link pair, we can satisfy the must-link constraint of (*d*_1_, *d*_3_) by assigning *d*_3_ to the same cluster as *d*_1_
  - If (*d*_1_, *d*_2_) and (*d*_1_, *d*_3_) are both cannot-link pairs, we can satisfy the cannot-link constraint of (*d*_1_, *d*_3_) by assigning *d*_3_ to a different cluster than *d*_1_.
- For (*d*_3_, *d*_4_)
  - If (*d*_1_, *d*_2_) and (*d*_3_, *d*_4_) are both must-link pairs, we can satisfy the must-link constraint of (*d*_3_, *d*_4_) by assigning (*d*_3_, *d*_4_) to the same cluster as (*d*_1_, *d*_2_).
  - If (*d*_1_, *d*_2_) is must-link pair and (*d*_3_, *d*_4_) is cannot-link pair, we can satisfy the cannot-link constraint of (*d*_3_, *d*_4_) by assigning *d*_3_ and *d*_4_ to different clusters.
  - If (*d*_1_, *d*_2_) is cannot-link pair and (*d*_3_, *d*_4_) is must-link pair, we can satisfy the must-link constraint of (*d*_3_, *d*_4_) by assigning *d*_3_ and *d*_4_ to the same cluster.
  - If (*d*_1_, *d*_2_) and (*d*_3_, *d*_4_) are both cannot-link pairs, we can satisfy the cannot-link constraint of (*d*_3_, *d*_4_) by assigning *d*_3_ and *d*_4_ to different clusters.

Thus, we can satisfy the constraint with the second priority in any case.

#### B.3 Constraint with Third Priority

For example, a constraint cannot be satisfied in the following case.

There are three data points, *d*_1_, *d*_2_, and *d*_3_. Let the nearest cluster for each data point be a, b, and c, respectively. Suppose there are three constraints with priorities as follows.

- Constraint with first priority: (*d*_1_, *d*_3_) is a cannot-link pair
- Constraint with second priority: (*d*_2_, *d*_3_) is a cannot-link pair
- Constraint with third priority: (*d*_1_, *d*_2_) is a must-link pair

After the data pair with the second priority is assigned, each data pair is assigned in the order *d*_1_ → *a, d*_2_ → *b, d*_3_ → *c*, so the constraint with the third priority cannot be satisfied in any way.

Thus, in our constrained k-means algorithm, constraints with the first and second priorities are guaranteed to be satisfied.
